# Acute Pulmonary Edema as a Manifestation of Thyroid Crisis From Hydatidiform Mole: A Case Report

**DOI:** 10.1155/crie/3431031

**Published:** 2026-08-24

**Authors:** Thaís Tiemi Yauti, Lin Yan, Fernanda Verza Azevedo, Paula Nina Shiroma, Cleomar Ana de Souza Valentim, Mariana Soares Dalla Mariga Jorgino

**Affiliations:** ^1^ Department of Internal Medicine, Faculty of Medicine of Jundiaí (FMJ), Jundiaí, São Paulo, Brazil, fmj.br; ^2^ Department of Public Health, Faculty of Medicine of Jundiaí (FMJ), Jundiaí, São Paulo, Brazil, fmj.br

**Keywords:** case report, hydatidiform mole, hyperthyroidism, pulmonary edema, thyroid crisis

## Abstract

**Objective:**

To report a clinical case of complete hydatidiform mole (CHM) with significant impact on thyroid function, associated with the development of acute pulmonary edema in the period following uterine evacuation.

**Case Report:**

A 46‐year‐old woman sought medical attention due to significant vaginal bleeding, with marked elevation of serum β‐hCG (greater than 300,000 mIU/mL), as well as transvaginal ultrasonography with imaging suggestive of HM. After uterine evacuation, the patient developed acute respiratory failure with dyspnea and chest pain, requiring intensive care admission and mechanical ventilation. Thyroid storm was diagnosed based on clinical presentation, laboratory findings, and Burch–Wartofsky criteria (50 points). Additional investigations excluded pulmonary embolism and primary thyroid disease. Removal of the HM resulted in decreased β‐hCG titers; however, normalization of serum thyroid hormone levels occurred only after pharmacological treatment.

**Conclusion:**

This case highlights the potential progression of hCG‐mediated thyrotoxicosis to thyroid crisis after uterine evacuation and emphasizes the importance of early recognition and treatment of severe hyperthyroidism in patients with HM. Early diagnosis and prompt management may help prevent thyroid crisis and cardiopulmonary complications. This case also demonstrates that acute pulmonary edema and transient cardiac dysfunction may occur as severe manifestations of thyroid crisis associated with gestational trophoblastic disease (GTD). Furthermore, close collaboration among obstetric, endocrine, and critical care teams is essential to optimize outcomes in complex presentations.

## 1. Introduction

Hydatidiform mole (HM) is the most common form of gestational trophoblastic disease (GTD) at a rate of one case per 1000–2000 pregnancies in western countries. One Brazilian study indicates, based on hospital care from a single center, a rate of one HM case per 215 pregnancies. GTD is a group of pathologies characterized by the abnormal proliferation of the placental trophoblastic epithelium. The primary biological marker of GTD is high levels of human chorionic gonadotropin (β‐hCG > 100,000 mUI/mL) [1]. The most frequent clinical symptom is vaginal bleeding, occurring in 84% of cases, often with a delayed menstrual cycle. Nausea and vomiting may also occur, sometimes refractory to treatment, especially in large moles with high β‐hCG levels. Maternal age, history of HM, viral infections, nutritional status, parity, consanguinity, and oral contraception are risk factors. Extremes of age present increased risk, and women over 40 years have a relative risk 10 times higher than women aged 22–40 years [2].

Thyroid‐stimulating hormone (TSH) and β‐hCG are glycoproteins with similar structures, sharing a common α‐subunit [3]. Due to this similarity, a significant elevation in serum β‐hCG levels (>200,000 mUI/mL) can sensitize TSH receptors and suppress TSH, leading to the overproduction of thyroid hormones (T3 and T4), but less than 10% of cases result in hyperthyroidism [2, 4, 5]. β‐hCG production by trophoblasts, however, is not inhibited by the increased thyroid hormones. The resulting hyperthyroidism can range from subclinical conditions to a thyroid crisis, which is clinically characterized by an exacerbation of hyperthyroidism symptoms (hyperpyrexia, gastrointestinal issues, tachycardia, and neurological alterations) and is considered a life‐threatening condition, mortality estimated to be 8%–25% [6]. However, more recent literature suggests the rate is 1.2%–3.6% in the U.S., which may be due to high‐quality, aggressive intensive care [7].

The transition from hyperthyroidism to thyroid crisis is often triggered by a systemic insult, such as surgery, trauma, infection, discontinuation of antithyroid medications, childbirth, or exposure to exogenous iodine or medications like amiodarone [7]. The diagnosis of thyroid crisis needs clinical criteria, so the Burch and Wartofsky scoring system [8] is used as a scoring system plus thyroid function tests are also obtained, which usually show high free T4 (FT4) or free T3 and low TSH. One should not wait for lab results before starting treatment [9]. This highlights the importance of observing patient signs and symptoms. The standard treatment for HM, uterine evacuation, can act as a precipitating factor for thyroid crisis, emphasizing the need for rigorous perioperative monitoring.

Clinical hyperthyroidism are rare complications of HM, and their early diagnosis is challenging, as hypermetabolism symptoms due to thyroid dysfunction can be mistaken for symptoms of molar pregnancy [6]. Without early diagnosis and treatment, the condition can progress to a thyroid crisis with life‐threatening multisystemic alterations. Therefore, improved perioperative monitoring, integrating clinical assessment and examination, is necessary to prevent serious complications.

This study describes a case of complete HM (CHM) associated with severe hCG‐mediated thyrotoxicosis that progressed to thyroid storm following uterine evacuation and was complicated by acute pulmonary edema, transient cardiac dysfunction, and respiratory failure requiring mechanical ventilation. Although thyrotoxicosis associated with the HM has been previously reported, this case illustrates the potential progression to thyroid storm with severe cardiopulmonary manifestations, including acute pulmonary edema, transient cardiac dysfunction, and respiratory failure requiring mechanical ventilation.

## 2. Case Report

A 46‐year‐old mixed‐race woman with a BMI of 22.7, no significant past medical history, and no relevant family history (gravida 2, para 1) was admitted to the gynecology and obstetrics service with complaints of vaginal bleeding and lower abdominal pain. She had irregular menstrual cycles and had noticed abdominal enlargement 1 week prior. Her serum β‐hCG measured over 300,000 mUI/mL (the reference for non‐pregnant women is up to 4 mUI/mL). She was scheduled for her first prenatal visit when she developed crampy abdominal pain and significant vaginal bleeding with clots, along with nausea and vomiting. The gestational age was estimated at 8 weeks and 5 days based on her last menstrual period, as no ultrasound had been performed. Her previous pregnancy was 15 years ago with no complications, resulting in a live vaginal birth (Table 1).

**Table 1:** Chronological timeline of clinical events, June 2024.

Clinical stage	Clinical event
Initial presentation	Vaginal bleeding, lower abdominal pain, nausea, vomiting, β‐hCG >300,000 mIU/mL, suppressed TSH, elevated FT4, and ultrasound findings suggestive of hydatidiform mole
First uterine evacuation	Curettage and manual intrauterine aspiration performed; significant intraoperative bleeding requiring volume expansion and red blood cell transfusion
Early postoperative period	Chest radiograph demonstrated bilateral pleural effusion; patient remained clinically stable and was discharged with propranolol
Readmission	Sudden dyspnea, chest tightness, diaphoresis, pallor, and acute respiratory failure requiring intubation and semi‐intensive care admission
Diagnostic investigation	Chest CT excluded pulmonary embolism; echocardiogram demonstrated heart failure, mitral insufficiency, and pulmonary hypertension; Burch–Wartofsky score of 50 points supported thyroid storm diagnosis
Thyroid storm treatment	Propylthiouracil, propranolol, and hydrocortisone initiated with progressive clinical and biochemical improvement
Clinical recovery	Successful extubation, improvement of cardiac function, normalization of ejection fraction, and hospital discharge
Follow‐up	Persistent uterine enlargement and abnormal ultrasound findings suggestive of retained molar tissue
Second uterine evacuation	Repeat uterine evacuation performed without complications
Ongoing surveillance	Serial β‐hCG, TSH, and FT4 monitoring due to the risk of gestational trophoblastic neoplasia

*Note: Source*: prepared by the authors.

**Figure 1:**
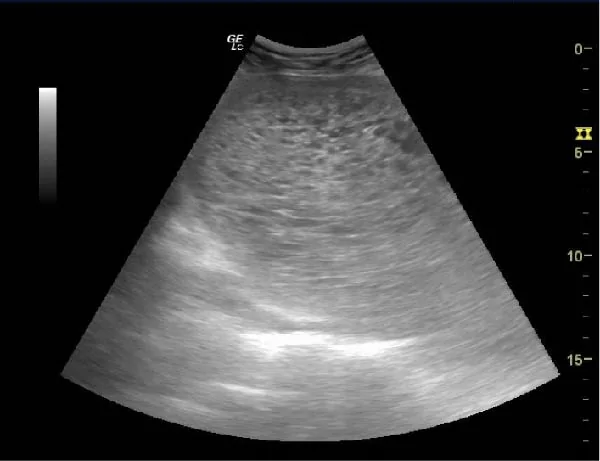
Transvaginal ultrasound with evidence of hydatidiform mole, just before the first curettage.

Upon physical examination, she was eupneic and tachycardic (120 bpm), with a blood pressure of 130/96 mmHg. Her uterine height was 25 cm. A speculum exam showed an epithelialized cervix with a small amount of bloody content. A vaginal exam revealed a fibroelastic posterior cervix, pervious to one finger breadth, painful on mobilization, with a free adnexa and a uterus enlarged for her gestational age. Laboratory tests showed normal platelet values (225,000), altered hemoglobin (9.5 mg/dL) and hematocrit (27.8%), a slight increase in leukocytes, TSH below 0.01 mUI/mL, and FT4 of 3.29 ng/dL, in addition to β‐hCG greater than 300,000 mUI/mL (values above 100,000 mUI/mL suggest GTD). A bedside transvaginal ultrasound (TVUS) was performed, showing images suggestive of a HM (Figure 1).

**Figure 2:**
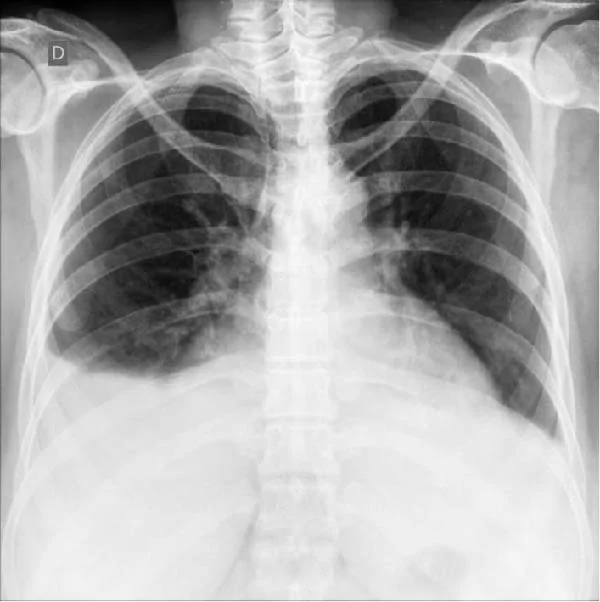
Posteroanterior chest radiograph with evidence of bilateral pleural effusion, 1 day after the first curettage.

The patient was taken to the operating room on the same day for curettage and manual intrauterine aspiration (MIA). Significant bleeding occurred during the procedure, requiring volume expansion with lactated Ringer’s solution and a red blood cell concentration transfusion. A chest X‐ray performed the day after the procedure (Figure 2) showed bilateral pleural effusion with a right‐sided predominance. However, as the patient did not have respiratory compromise during her hospitalization, she was discharged after 4 days with scheduled outpatient follow‐up and a prescription for propranolol 20 mg twice daily. The anatomopathological examination of the removed intrauterine content showed spongy fragments with cystic formations resembling grape clusters, consistent with CHM and no signs of malignancy.

Three days after discharge, the patient was admitted to the emergency service with sudden onset dyspnea, chest tightness, diaphoresis, and pallor. Her physical exam showed tachycardia (116 bpm) and crackles on pulmonary auscultation were noted. Due to acute respiratory failure, noninvasive ventilation (NIV) was attempted twice without improvement. She was then intubated and admitted to the semi‐intensive care unit. Based on her clinical presentation and laboratory findings, the patient scored 50 points on the Burch and Wartofsky scale [8] (Table 2), indicating a high possibility of a thyroid crisis.

**Table 2:** Burch–Wartofsky point scale score supporting the diagnosis of thyroid crisis, June 2024.

Parameter	Finding	Points
Temperature	<37.2°C	5
CNS dysfunction	Mild	10
Gastrointestinal dysfunction	Moderate	10
Tachycardia	116 bpm	10
Pulmonary edema	Present	15
Atrial fibrillation	Absent	0
Precipitating event	Surgery	10
Total		50

*Note: Source*: adapted from Burch and Wartofsky [8]. Interpretation: highly suggestive of thyroid storm.

A chest CT scan on admission excluded pulmonary thromboembolism but showed pleural and pericardial effusion, cardiomegaly, and pulmonary consolidation, suggestive of a pneumonic process. A transthoracic echocardiogram the next day identified heart failure with an ejection fraction (EF) of 49% (reference 50%–70%), moderate eccentric left ventricular hypertrophy, significant mitral insufficiency, and pulmonary hypertension.

Alternative diagnoses considered included pulmonary thromboembolism, pneumonia, transfusion‐related pulmonary injury, and primary cardiac disease. Pulmonary thromboembolism was excluded by computed tomography, while thyroid ultrasonography and negative TSH receptor antibody (TRAb) results argued against underlying primary thyroid disease. Although pulmonary consolidation raised the possibility of infection, the marked biochemical thyrotoxicosis, Burch–Wartofsky score of 50 points, and rapid response to antithyroid therapy strongly supported thyroid storm as the principal diagnosis [7, 8, 10].

Due to the high possibility of thyroid crisis, the patient was treated with propylthiouracil 100 mg every 4 h, propranolol 40 mg every 6 h orally, and hydrocortisone 100 mg every 8 h intravenously. The treatment was well tolerated, with good adherence and no reported adverse effects. After leaving the semi‐intensive care unit, lab measurements of β‐hCG, TSH, FT4, TRAb, and a thyroid ultrasound with Doppler were performed. Results showed β‐hCG 29,735.00 mUI/mL, TSH < 0.01 mUI/mL, FT4 1.14 ng/dL, and TRAb < 0.01 IU/L (reference 1.75 UI/L). The ultrasound showed minute spongiform nodules in the right thyroid lobe and a cyst in the left lobe, classified as TI‐RADS 2, indicating no primary thyroid disease.

After starting treatment, the patient’s condition improved clinically, and FT4 decreased from 3.29 ng/dL at diagnosis to 1.14 ng/dL after initiation of antithyroid therapy, representing an approximate 65% reduction. This biochemical improvement paralleled clinical recovery, including successful extubation and improvement of cardiac function. She was extubated on the second day and transferred to the medical ward on the fourth day. A new echocardiogram showed eccentric left ventricular hypertrophy, but the mitral insufficiency was now mild, and the EF was 60%. Both cardiac dysfunction and respiratory failure were attributed to the HM.

**Figure 3:**
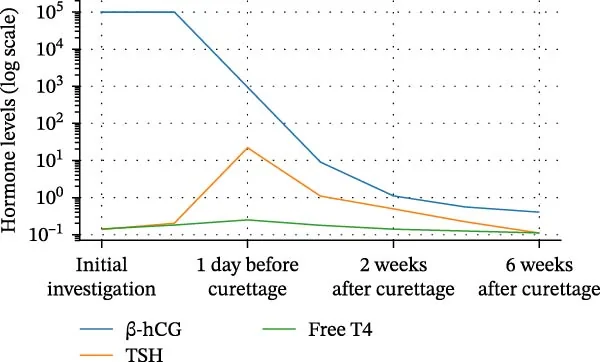
Patient’s serum β‐hCG, TSH, and FT4 values. *Source*: prepared by the authors.

After clinical stability, propranolol and hydrocortisone were discontinued. She was discharged after 17 days with instructions to reduce the propylthiouracil dose to 100 mg every 8 h, followed by a gradual weaning and weekly outpatient follow‐up. During subsequent outpatient follow‐up, she reported intermittent bleeding, and a physical exam revealed uterine enlargement, palpable 3 cm above the pubic symphysis. A new ultrasound showed endometrial thickening suggestive of molar pregnancy remnants. She was then hospitalized for a second uterine evacuation, which was performed without any complications. The patient continues outpatient follow‐up for β‐hCG, TSH, and FT4 evaluation (Figure 3) to monitor for the possibility of gestational trophoblastic neoplasia (GTN), as her β‐hCG levels increased by more than 10% in three measurements over 2 weeks after successive curettages (FIGO 2000) [11]. Throughout this process, the patient noted a profound sense of relief following the stabilization of her condition and expressed optimism about her long‐term recovery.

## 3. Discussion

This case illustrates the progression from β‐hCG‐mediated hyperthyroidism to clinically significant thyrotoxicosis and, ultimately, to thyroid crisis following uterine evacuation, complicated by acute pulmonary edema and transient cardiac dysfunction requiring intensive care support.

In this case, the patient’s CHM with high β‐hCG levels led to thyroid stimulation, increased FT4, and TSH suppression without negative feedback on β‐hCG. Serial T3 measurements were not available during follow‐up; however, current guidelines do not recommend T3 monitoring as a prognostic marker in thyroid crisis [10]. Uterine evacuation with MIA, the standard treatment, may have been a precipitating factor for the conversion of hyperthyroidism into a thyroid crisis [7]. Sharma et al. [12] emphasized the importance of using the Burch–Wartofsky score [8] to aid in early diagnosis. In their study, a score of 30 points at admission led to intensive care for crisis management, followed by curettage of the mole [12].

To further characterize the severity of the patient’s condition, retrospective application of the obstetric early warning score (OEWS) [13], sequential organ failure assessment (SOFA) [14], and acute physiology and chronic health evaluation II (APACHE II) [15] demonstrated significant physiological deterioration requiring intensive care support. Detailed score calculations are provided in Tables S1–S3.

The patient returned to the hospital 3 days after curettage with symptoms of respiratory failure, diaphoresis, and pallor. Alternative causes of acute respiratory deterioration, including pulmonary embolism, pneumonia, transfusion‐related pulmonary injury, and primary cardiac disease, were considered. Pulmonary embolism was excluded by chest CT, while thyroid ultrasound, together with negative TRAb results, argued against primary thyroid disease. The combination of marked biochemical thyrotoxicosis, a Burch–Wartofsky [8] score of 50 points, and rapid clinical improvement following antithyroid therapy strongly supported the diagnosis of thyroid crisis. After the initiation of antithyroid therapy, FT4 decreased from 3.29 to 1.14 ng/dL, representing an approximate 65% reduction. This biochemical improvement paralleled the patient’s clinical recovery, including successful extubation and improvement of cardiac function.

Desai et al. [16] reported that approximately 16% of hospitalizations for hyperthyroidism evolve into a thyroid crisis, with substantially increased mortality compared with uncomplicated hyperthyroidism. Cardiovascular complications, arrhythmias, and multiple organ dysfunction are major contributors to adverse outcomes [6]. Our patient developed acute pulmonary edema and transient cardiac dysfunction but improved rapidly following the initiation of antithyroid therapy and supportive care.

The patient was treated with propranolol, hydrocortisone, and propylthiouracil according to established principles of thyroid crisis management [2, 10, 12]. Current guidelines recommend higher initial doses of antithyroid medication in severe hyperthyroidism and thyroid crises [10].

The possibility of a thyroid storm was not initially recognized, and PTU was prescribed at a dose of 100 mg every 8 h, which is below the recommended regimen for severe hyperthyroidism or thyroid storm. Current guidelines from the American Thyroid Association and the American Association of Clinical Endocrinologists [10] recommend higher initial dosing (500–1000 mg loading dose followed by 200–250 mg every 4 h) in such scenarios.

From an obstetrical standpoint, maternal stabilization prior to surgical intervention is a key principle. In patients with GTD and significant thyrotoxicosis, current recommendations support beta‐adrenergic blockade and, when clinically feasible, initiation of antithyroid therapy before uterine evacuation [2, 10]. As uterine evacuation may act as a precipitating factor for thyroid storm, the absence of preoperative control of thyrotoxicosis may have contributed to the clinical deterioration observed in our patient [7, 12].

Similar to our case, the study by Frasik et al. [17] highlights that markedly elevated β‐hCG levels are the main factor responsible for exacerbated thyroid stimulation in pregnant women with GTD, intensifying hyperthyroidism symptoms and potentially leading to a thyroid crisis without early intervention. Our patient developed additional complications, such as acute pulmonary edema and cardiac dysfunction, which worsened her prognosis.

Persistence of high β‐hCG levels may indicate the evolution of HM to GTN. Up to 50% of molar pregnancy cases are followed by GTN, with 15% of patients who had complete molar pregnancy potentially presenting persistent trophoblastic disease with invasion [9]. That makes patient follow‐up in specialized services necessary, even with anatomopathological examination indicating benign disease.

Although this case report has limitations regarding confounding factors and does not allow for the generalization of symptoms, it enables the description of possible clinical conditions and outcomes associated with the pathology, which contributes to its management and diagnosis. An additional limitation is the absence of serial T3 measurements during follow‐up.

## 4. Conclusion

This case report illustrates how excessive β‐hCG levels associated with HM can induce severe thyrotoxicosis and progression to thyroid storm, resulting in life‐threatening complications such as acute pulmonary edema and transient cardiac dysfunction. Early thyroid function assessment should be considered in patients with markedly elevated β‐hCG levels, particularly before uterine evacuation. Early recognition, multidisciplinary management, and adequate preoperative stabilization may reduce the risk of severe cardiopulmonary complications.

## Acknowledgments

The authors used ChatGPT (OpenAI) solely for English language editing, grammar correction, and readability improvement. No AI tools were used for study design, data analysis, interpretation of results, or generation of scientific conclusions. We thank everyone involved in this work for their dedication and commitment. We also thank our families for supporting us during the preparation of this study.

## Funding

This case report received no specific grant from any funding agency in the public, commercial, or not‐for‐profit sectors.

## Disclosure

All scientific content and final manuscript revisions were performed and approved by the authors, who take full responsibility for the accuracy and integrity of the work. All authors have read and approved the final version of the manuscript. Mariana Soares Dalla Mariga Jorgino had full access to all study data and take responsibility for the data integrity and accuracy.

## Ethics Statement

The study was approved by the Research Ethics Committee of Jundiaí Medical School (CAAE: 82510824.4.0000.5412, approval number 7.285.408). Written informed consent was obtained from the patient for publication of this case report and any accompanying images or clinical information in accordance with institutional and ethical standards.

## Conflicts of Interest

The authors declare no conflicts of interest.

## Supporting Information

Additional supporting information can be found online in the Supporting Information section.

## Supporting information


**Supporting Information** Table S1: Obstetric early warning score (OEWS). Table S2: Sequential organ failure assessment (SOFA). Table S3: Acute physiology and chronic health evaluation II (APACHE II). CARE case report guidelines.

## Data Availability

The data of this study are available upon request from the corresponding author.
