# Supplementary material for: Acute Pulmonary Edema as a Manifestation of Thyroid Crisis From Hydatidiform Mole: A Case Report
Source: Case Rep Endocrinol. 2026 Aug 24;2026:3431031. doi: 10.1155/crie/3431031 (PMC13504243; doi:10.1155/crie/3431031)
Supplement: Supplementary file 1 [file CRIE-2026-3431031-s001.zip › 3431031/3431031.f1.docx]

**Supplementary Table S1. Obstetric Early Warning Score (OEWS) applied retrospectively to the patient**

| **Parameter** | **Value in Case Report** | **Points** |
| --- | --- | --- |
| Respiratory rate | Acute respiratory failure requiring ventilatory support | 3 |
| Oxygen saturation  O2% | Need for non-invasive ventilation followed by endotracheal intubation | 3 |
| Heart rate | 116 bpm | 1 |
| Blood pressure | 130/96 mmHg (recorded at first admission) | 0 |
| Level of consciousness | Mild central nervous system dysfunction | 0 |
| Temperature | <37.0ºC | 0 |
| **Total score** |  | **7** |

Interpretation: High-risk score indicating the need for urgent clinical intervention.

Source: Prepared by the authors based on the OEWS scale [13].

**Supplementary Table S2. Sequential Organ Failure Assessment (SOFA) score applied retrospectively to the patient**

| **System** | **Clinical data from Case Report** | **Points** |
| --- | --- | --- |
| Respiratory | Acute respiratory failure requiring non-invasive ventilation (NIV) failure and subsequent endotracheal intubation | 2 |
| Coagulation | Platelet count 225,000/mm³ | 0 |
| Liver | Bilirubin levels were not specifically provided in the report | 0 |
| Cardiovascular | Tachycardia (116 bpm) and heart failure with an ejection fraction (EF) of 49% | 1 |
| Neurological | Mild central nervous system dysfunction | 2 |
| Renal | No renal dysfunction reported | 0 |
| **Total score** |  | **5** |

Interpretation: Early organ dysfunction, predominantly involving respiratory and neurological systems.

*Source: Prepared by the authors based on the SOFA score [14].*

**Supplementary Table S3. Acute Physiology and Chronic Health Evaluation II (APACHE II) score applied retrospectively to the patient**

| **Variable** | **Value from Case Report** | **Points** |
| --- | --- | --- |
| Age | 46 years | 2 |
| Temperature | 37.2ºC | 0 |
| Mean arterial pressure | Approximately 107 mmHg | 0 |
| Heart rate | 116 bpm | 2 |
| Respiratory status | Acute respiratory failure requiring intubation | 3 |
| Oxigenation | Acute pulmonary edema | 3 |
| Hematocrit | 27.8% | 2 |
| Leukocyte count | Mild leukocytosis | 1 |
| Chornic health status | No known comorbidade | 0 |
| **Total score** |  | **13** |

Interpretation: Moderate disease severity with significant risk of clinical deterioration.

*Source: Prepared by the authors based on the APACHE II score [15].*
