# Supplementary material for: Acute Pulmonary Edema as a Manifestation of Thyroid Crisis From Hydatidiform Mole: A Case Report
Source: Case Rep Endocrinol. 2026 Aug 24;2026:3431031. doi: 10.1155/crie/3431031 (PMC13504243; doi:10.1155/crie/3431031)
Supplement: Supplementary file 1 [file CRIE-2026-3431031-s001.zip › 3431031/3431031.f2.pdf]

CARE Checklist of information to include when writing a case report

CARE case report guidelines

BY-NC-ND

Topic	Item	Checklist item description	Reported on Line
Title	1	The diagnosis or intervention of primary focus followed by the words "case report"	1-2
Key Words	2	2 to 5 key words that identify diagnoses or interventions in this case report, including "case report"	36-37
Abstract (no references)	3a	Introduction: What is unique about this case and what does it add to the scientific literature?	14-16
	3b	Main symptoms and/or important clinical findings	17-22
	3c	The main diagnoses, therapeutic interventions, and outcomes	22-26
	3d	Conclusion—What is the main "take-away" lesson(s) from this case?	27-35
Introduction	4	One or two paragraphs summarizing why this case is unique (**may include references**)	39-89
Patient Information	5a	De-identified patient specific information.	92-93
	5b	Primary concerns and symptoms of the patient.	94-96
	5c	Medical, family, and psycho-social history including relevant genetic information	92-93
	5d	Relevant past interventions with outcomes	101-102
Clinical Findings	6	Describe significant physical examination (PE) and important clinical findings.	107-111
Timeline	7	Historical and current information from this episode of care organized as a timeline	104
Diagnostic Assessment	8a	Diagnostic testing (such as PE, laboratory testing, imaging, surveys).	112-201
	8b	Diagnostic challenges (such as access to testing, financial, or cultural)	211-213, 243-257
	8c	Diagnosis (including other diagnoses considered)	158-159, 205-208
	8d	Prognosis (such as staging in oncology) where applicable	192-196
Therapeutic Intervention	9a	Types of therapeutic intervention (such as pharmacologic, surgical, preventive, self-care)	166-184
	9b	Administration of therapeutic intervention (such as dosage, strength, duration)	166-184
	9c	Changes in therapeutic intervention (with rationale)	185-188
Follow-up and Outcomes	10a	Clinician and patient-assessed outcomes (if available)	185-196
	10b	Important follow-up diagnostic and other test results	192-196
	10c	Intervention adherence and tolerability (How was this assessed?)	168-169
	10d	Adverse and unanticipated events	168-169
Discussion	11a	A scientific discussion of the strengths AND limitations associated with this case report	272-286
	11b	Discussion of the relevant medical literature **with references**.	215-265
	11c	The scientific rationale for any conclusions (including assessment of possible causes)	243-259
	11d	The primary "take-away" lessons of this case report (without references) in a one paragraph conclusion	279-286
Patient Perspective	12	The patient should share their perspective in one to two paragraphs on the treatment(s) they received	196-198
Informed Consent	13	Did the patient give informed consent? Please provide if requested	Yes ☒ No ☐
